# Foliar Application of Oil Palm Wood Vinegar Enhances *Pandanus amaryllifolius* Tolerance under Drought Stress

**DOI:** 10.3390/plants12040785

**Published:** 2023-02-09

**Authors:** Muhammad Asyraf Mohd Amnan, Wee Fei Aaron Teo, Wan Mohd Aizat, Fiqri Dizar Khaidizar, Boon Chin Tan

**Affiliations:** 1 Centre for Research in Biotechnology for Agriculture (CEBAR), Universiti Malaya, Kuala Lumpur 50603, Malaysia; 2 Institute of Systems Biology (INBIOSIS), Universiti Kebangsaan Malaysia, Bangi 43600, Malaysia

**Keywords:** agriculture, antioxidant enzymes, biostimulants, drought stress, wood vinegar, drought-responsive genes

## Abstract

Drought stress severely threatens plant growth, yield and survivability. Wood vinegar, formed by the condensation of smoke produced during biochar production, has been shown to promote plant growth and enhance stress tolerance. They have now been recognized as a sustainable alternative and are frequently used exogenously to support plants coping with environmental stress. This study aimed to evaluate the efficacy of oil palm wood vinegar (OPWV) in mitigating the adverse effects of drought stress on *Pandanus amaryllifolius*. The optimal concentrations and frequencies of OPWV application were determined before the drought treatment. The results showed that the imposed drought stress negatively affected the plant growth parameters but applying OPWV at 1:500 dilution at 3-day intervals for 12 days increased its tolerance. These include increased leaf relative water content, root-to-shoot ratio, relative stem circumference, chlorophyll pigments and antioxidant enzyme activities. In contrast, the drought-stressed plants treated with OPWV showed decreased relative electrolyte leakage, hydrogen peroxide, proline, malondialdehyde, and enhanced drought-responsive gene expressions, such as *HSP70*, *GAPDH*, and *Thau*, while *ENO* and *β-Fruc* were reduced. These biostimulatory effects of OPWV might be due to several antioxidant compounds, such as anthranilic acid, tetrasiloxane, syringol, guaiacol, and catechol. Altogether, our results showed the effectiveness of OPWV in alleviating the adverse effects of drought stress, and as such, OPWV could be potentially applied in agriculture.

## 1. Introduction

Climate change has increased the occurrence, intensity, and complexity of the multifactorial combination of stresses, affecting agricultural production. For example, the flooding event in Malaysia in 2006 resulted in a loss of USD 18.9 million in agriculture damages [1], while drought has caused USD 30 billion in global crop yield losses over the past decade [2]. Among the environmental stresses, drought stress is a significant threat to crop production. It affects about 55 million people’s socioeconomic activity globally [3].

Drought stress negatively affects plant growth and development. When plants are exposed to drought stress, their biochemical and metabolic activities are disrupted due to the limited water supply. This hinders some critical processes, such as photosynthesis, nutrient acquisition, transportation of minerals and translocation, leading to excessive reactive oxygen species (ROS) accumulation [4]. Although plants have defense machinery to protect themselves against oxidative stress damages, exploring innovative and sustainable interventions to mitigate the drought stress effects on plant growth is indispensable.

Biostimulants are any substance or microorganism applied to plants, aiming to increase nutrition efficiency, environmental stress tolerance and/or crop quality traits [5]. They have gained much interest because of their ability to stimulate growth and mitigate stress-induced limitations. Moreover, biostimulants have been recognized as a sustainable alternative to support plants coping with environmental stress. For instance, seaweed extract [6] and chitosan [7] have enhanced the plant’s yield and tolerance against drought stress. In addition, the exogenous application of wood vinegar [8] and smoked water [9] elevated the drought tolerance of rice and cowpea, respectively.

Wood vinegar or pyroligneous acid is a reddish-brown translucent liquid produced as a by-product of pyrolysed wood heated from 200 °C to 450 °C under low oxygen conditions. The accumulated smoke from the kiln is then channeled into a condensation tube to form a condensed smoked liquid [10]. Wood vinegar consists of many beneficial compounds. For example, wood vinegar derived from lychee wood consists of three major components, which are 2,6-dimethoxyphenol (syringol) and 2-methoxyphenol (guaiacol), and 3,5-dimethoxy-4-hydroxytoluene (pyrogallol) [11]. These compounds were reported for their ability as an antioxidant to scavenge ROS [12,13,14]. Previous studies reported that wood vinegar could be used as a biostimulant to promote plant performance and growth [15,16]. Additionally, Wang et al. reported that wood vinegar enhanced wheat tolerance against drought by increasing abscisic acid accumulation, antioxidant activities and reducing ROS [17]. Despite its potential, applying wood vinegar to mitigate the adverse effect of environmental stresses is scarce. Therefore, this study explored the benefits of wood vinegar produced from oil palm kernel shells on plant growth and development.

*Pandanus amaryllifolius*, a member of the botanical family Pandanaceae, is a tropical plant species commonly referred to as fragrant screw pine or pandan [18]. This species is widely cultivated in Southeast Asia. The leaves of *P. amaryllifolius* are commonly used as flavoring agents, natural colorants, and in traditional medicine [18]. Phytochemical analysis of *P. amaryllifolius* leaves has revealed the presence of phenolic compounds, such as gallic acid and cinnamic acid, which have been shown to inhibit the growth of breast cancer cells in laboratory studies [19]. Additionally, the leaf extract of *P. amaryllifolius* has been found to have potentially beneficial effects on metabolic syndromes, such as weight gain and blood pressure.

We previously determined the effects of mild and severe drought stress on morphological, biochemical, and proteomics changes of *P. amaryllifolius* [18]. Our results showed that *Pandanus* plants could endure up to 10 days of drought in clay silt loam soil. Since wood vinegar may act by priming the treated crops against environmental stresses, we hypothesized that the exogenous application of oil palm wood vinegar (OPWV) would improve *P. amaryllifolius* growth and enhance its tolerance to drought stress. To address our hypotheses, we investigated the responses of *P. amaryllifolius* plants under well-watered and drought-stress conditions with or without OPWV treatment. In addition, we also determined the optimal dilution factors and application frequencies of OPWV and its compound profile. The novelty of our study is that OPWV has not been explored to enhance plant growth and stress tolerance. Since OPWV is made from the waste products of oil palm trees, this makes it a more sustainable and eco-friendly option compared to other wood vinegar. Additionally, OPWV may have unique chemical properties and composition compared to wood vinegar made from other types of wood, which could make it useful for different applications.

## 2. Results

### 2.1. Different Oil Palm Wood Vinegar (OPWV) Dilution Factors and Application Frequencies on Pandanus amaryllifolius Growth

**Figure 1:**
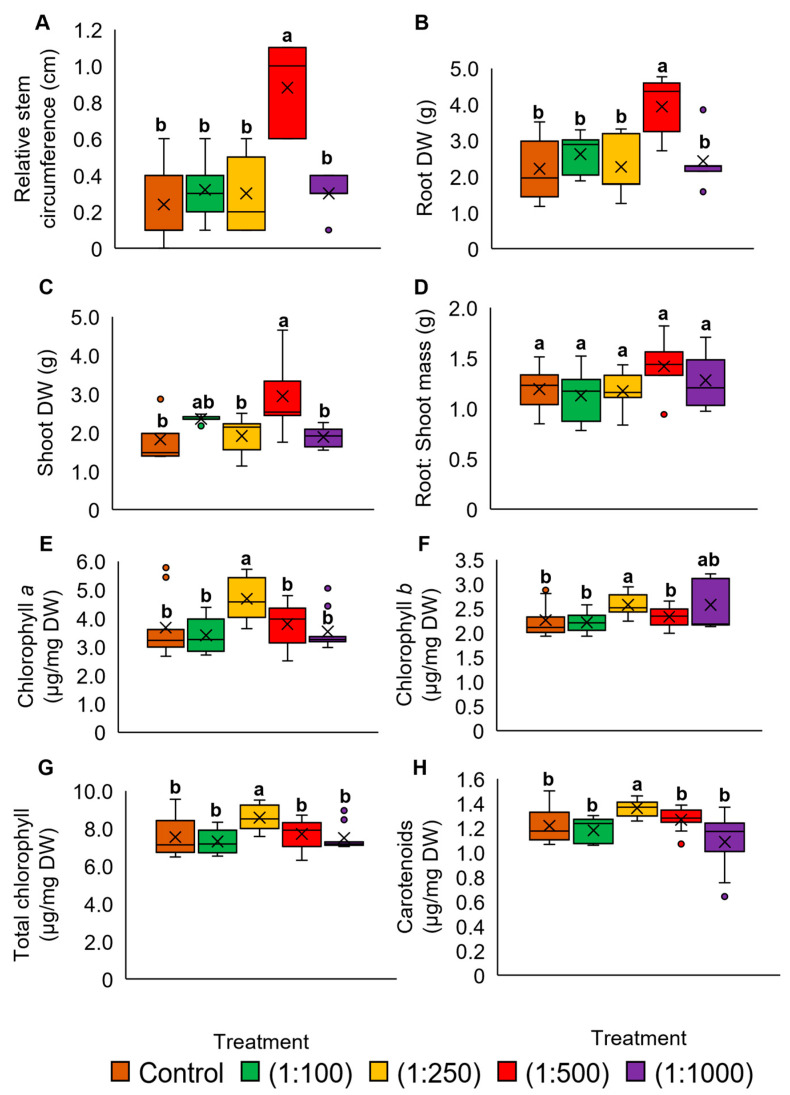
The plant morphological and pigment analyses of the *Pandanus amaryllifolius* treated with different dilution factors of oil palm wood vinegar (OPWV). (**A**) Relative stem circumference. (**B**) Root dry weight (DW). (**C**) Shoot DW. (**D**) The root-to-shoot ratio. (**E**) Chlorophyll *a* (µg mg^−1^ DW). (**F**) Chlorophyll *b* (µg mg^−1^ DW). (**G**) Total chlorophyll (µg mg^−1^ DW). (**H**) Carotenoids (µg mg^−1^ DW). The letter labelled on the mean value indicates a significant level between treatments based on the one-way ANOVA, followed by the post hoc Tukey test when its *p*-value < 0.05. The letter ‘a’ above the bars indicates the highest value, followed by ‘ab’ and ‘b’. The (×) labelled in the box plot indicates the mean value of the treatment, while the (•) refers to the outlier value of the replicates.

*P. amaryllifolius* plants were sprayed with OPWV at different dilution factors. All treated plants did not show visible differences (Figure S1A). However, the relative stem circumference and root dry weight (DW) of plants treated with OPWV at 1:500 dilution were significantly higher than other treatments (Figure 1A,B). Although this treatment also produced a higher shoot DW than others, the shoot-to-root ratio of all treatments showed no significant changes (Figure 1C,D). Regarding chlorophyll contents, although the 1:250 OPWV treatment increased the chlorophyll contents (Figure 1E–H), OPWV at 1:500 dilution was chosen for the subsequent experiment, as it showed enhanced stem circumference and biomass.

**Figure 2:**
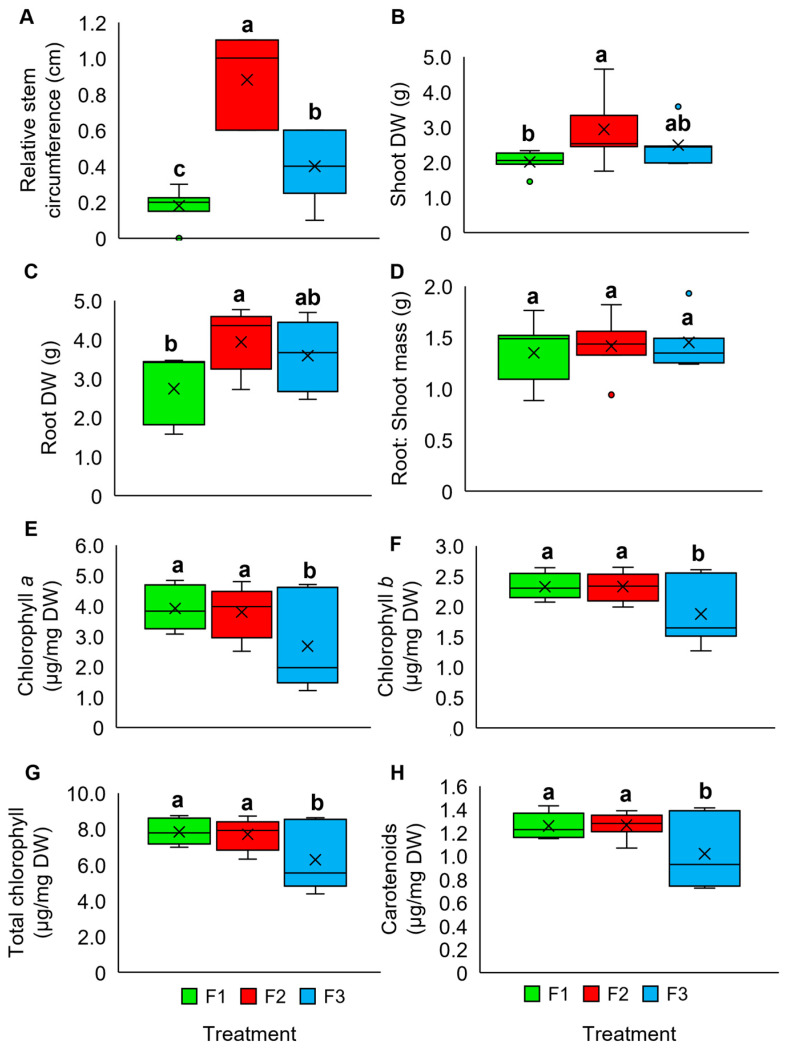
The plant morphological and pigment analyses of the *Pandanus amaryllifolius* were subjected to different application frequencies. F1: applied once at 6-day intervals, F2: applied once at 3-day intervals, and F3: applied once at 1-day intervals. (**A**) Relative stem circumference. (**B**) Shoot dry weight (DW). (**C**) Root DW. (**D**) The root-to-shoot ratio (**E**) Chlorophyll *a* (µg mg^−1^ DW). (**F**) Chlorophyll *b* (µg mg^−1^ DW). (**G**) Total chlorophyll (µg mg^−1^ DW). (**H**) Carotenoids (µg mg^−1^ DW). The letter labelled on the mean value indicates a significant level between treatments based on the one-way ANOVA, followed by the post hoc Tukey test when its *p*-value < 0.05. The letter ‘a’ above the bars indicates the highest value, followed by ‘ab’, ‘b’, and ‘c’. The (×) labelled in the box plot indicates the mean value of the treatment, while the (•) refers to the outlier value of the replicates.

Different application frequencies of the diluted OPWV at 1:500 were evaluated. In general, plants treated with OPWV at different frequencies showed no visible differences (Figure S1B). However, F2-treated plants showed the highest mean relative stem circumference (0.9 cm) and shoot (2.9 g) and root DW (3.9 g) (Figure 2A–C). In contrast, the root-to-shoot ratio for all treatments was not significantly different (Figure 2D). The plants treated with F1 and F2 frequencies showed a higher accumulation of pigment constituents than those treated with F3 (Figure 2E–H). Therefore, a combination of the 1:500 dilution and F2 application frequency was selected for the subsequent experiments.

### 2.2. Oil Palm Wood Vinegar (OPWV) Improved Drought-Stressed Pandanus amaryllifolius

**Figure 3:**
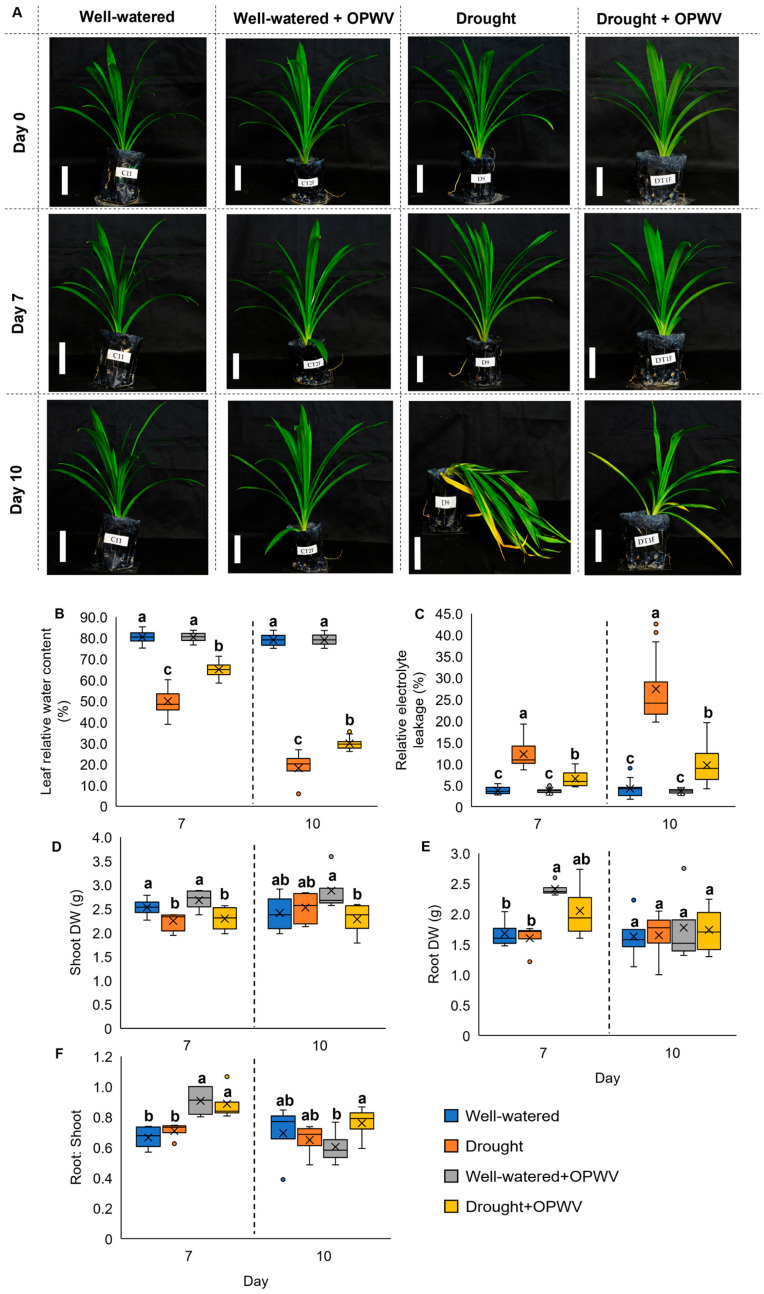
The morphological analysis of the well-watered and drought-stressed *Pandanus amaryllifolius* plants treated with or without OPWV for 7 and 10 days. (**A**) Representative photographs of *P. amaryllifolius* under different conditions. Bar = 10 cm. (**B**) Leaf relative water content (LRWC). (**C**) Leaf relative electrolyte leakage (REL). (**D**) The shoot dry weight (DW). (**E**) Root DW. (**F**) Root-to-shoot mass ratio. The letter labelled on the mean value indicates a significant level between treatments based on the one-way ANOVA, followed by the post hoc Tukey test when its *p*-value < 0.05. The letter ‘a’ above the bars indicates the highest value, followed by ‘ab’, ‘b’, and ‘c’. The (**×**) labelled in the box plot indicates the mean value of the treatment, while the (•) refers to the outlier value of the replicates.

To determine the biostimulant effects of OPWV against a drought environment, the morphological changes of plants subjected to water withholding for 7 and 10 days were recorded (Figure 3A). The 7-day drought-stressed plants without OPWV treatment showed notable stress symptoms, such as wilting and leaf folding. However, the plants showed more severe effects when prolonged drought stress conditions to 10 days. These include losing their structural turgor, and some completely collapsed (Figure 3A). In contrast, plants with OPWV treatment could sustain their leaf structure, showing minimal wilting and leaf folding despite water withholding for 7 days, and retain their turgor pressure, sustaining their upright structure (Figure 3A).

The plant leaf relative water content (LRWC) and relative electrolyte leakage (REL) were analyzed to investigate the degree of water loss and cell damage within the plant cells. The well-watered plants, with or without OPWV treatment, showed more than 80% LRWC for both days 7 and 10 (Figure 3B). However, a significant 50% and 18% decrease was observed in the drought-stressed plants without OPWV treatment on days 7 and 10, respectively. On the contrary, despite drought stress, OPWV-treated *P. amaryllifolius* retained 65% and 30% LRWC on days 7 and 10, respectively. Regarding REL, the well-watered plants, with or without OPWV treatment, showed the lowest REL (4%) on days 7 and 10 (Figure 3C). In contrast, the drought-stressed plants without OPWV treatment recorded the highest REL of 12% and 27% on days 7 and 10, respectively, followed by the OPWV-treated drought-stressed plants with 7% and 10% REL on days 7 and 10, respectively.

The DW of shoots and roots and the root-to-shoot ratio of the plant samples were recorded. The well-watered and OPWV-treated plants showed higher shoot DW than the drought-stressed plants on day 7, although it was not significantly different on day 10 (Figure 3D). On the other hand, the OPWV-treated plants showed increased root DW on day 7 (Figure 3E). Similar to shoot DW, all treatments showed no significant difference on day 10 (Figure 3E). However, having a higher root and shoot DW, it is no surprise that the OPWV-treated plants recorded the highest root-to-shoot ratio on day 7 (Figure 3F).

**Figure 4:**
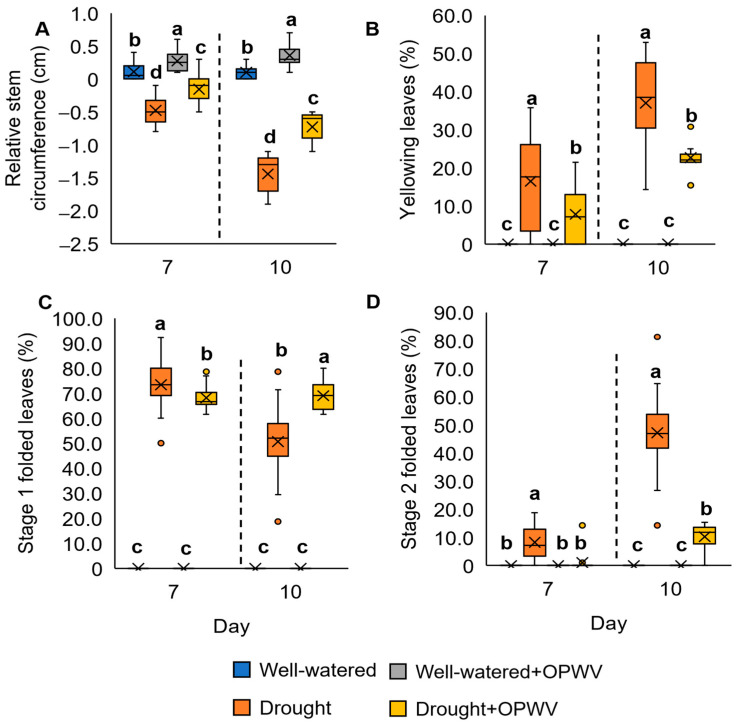
The stem and leaf morphological analyses of the *Pandanus amaryllifolius* treated with 1:500 diluted OPWV at 3-day intervals under well-watered and drought stress conditions for 7 and 10 days. (**A**) The relative stem circumference. (**B**) The percentage of yellowing leaves. (**C**) The percentage of Stage 1 leaf folding. (**D**) The percentage of Stage 2 leaf folding. The letter labelled on the mean value indicates a significant level between treatments based on the one-way ANOVA, followed by the post hoc Tukey test when its *p*-value < 0.05. The letter ‘a’ above the bars indicates the highest value, followed by ‘b’, ‘c’, and ‘d’. The (×) labelled in the box plot indicates the mean value of the treatment, while the (•) refers to the outlier value of the replicates.

The highest relative stem circumference was recorded in the well-watered *P. amaryllifolius* treated with OPWV for days 7 (0.3 cm) and 10 (0.4 cm), followed by well-watered (0.1 cm for both 7 and 10 days), OPWV-treated drought-stressed (−0.2 and −0.7 cm for 7 and 10 days, respectively), and drought-stressed samples (−0.5 and −1.4 cm for 7 and 10 days, respectively) (Figure 4A).

The percentage of leaf yellowing was analyzed based on the leaf color reference (Figure S2E). Both OPWV-treated and non-treated well-watered samples showed no yellowing symptoms (Figure 4B). On the contrary, the drought-stressed *P. amaryllifolius* showed 16% and 37% yellowing leaves on days 7 and 10, respectively (Figure 4B). In comparison, the percentage of leaf yellowing for the OPWV-treated samples was lower, 8% and 23% on days 7 and 10, respectively.

The percentage of leaves folding was determined according to the references (Figure S2D). After 7 days of drought stress, the plants showed a higher percentage of stage 1 folding (73%) compared to the OPWV-treated drought-stressed samples (68%) (Figure 4C). However, the 10-day drought-stressed plants showed a lower percentage of stage 1 folding (51%) than the OPWV-treated samples (69%) (Figure 4C). This result indicates the shift of leaf folding severity from Stage 1 to Stage 2. On the other hand, the OPWV-treated drought-stressed samples maintained the Stage 1 leaf folding and had lower Stage 2 leaf folding than those without OPWV treatment (Figure 4D).

### 2.3. Leaf Pigment Constituents in Pandanus amaryllifolius in Response to OPWV

**Figure 5:**
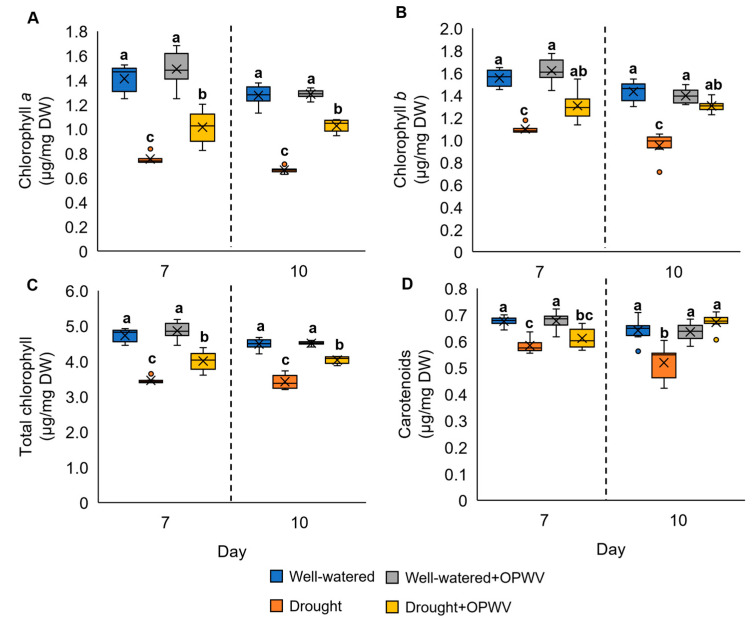
The pigmentation constituents of the well-watered and drought-stressed plants with or without OPWV for 7 and 10 days. (**A**) Chlorophyll *a* (µg mg^−1^ DW). (**B**) Chlorophyll *b* (µg mg^−1^ DW). (**C**) Total chlorophyll (µg mg^−1^ DW). (**D**) Carotenoids (µg mg^−1^ DW). The letter labelled on the mean value indicates a significant level between treatments based on the one-way ANOVA, followed by the post hoc Tukey test when its *p*-value < 0.05. The letter ‘a’ indicates the highest value, followed by ‘ab’, ‘b’, and ‘c’. The (×) labelled in the box plot indicates the mean value of the treatment, while the (•) refers to the outlier value of the replicates.

In this study, the drought-stressed *P. amaryllifolius* exhibited a significant reduction in chlorophyll *a*, chlorophyll *b*, total chlorophyll, and carotenoids on days 7 and 10 compared to well-watered samples (Figure 5A–D). In addition, when treated with OPWV, the pigmentation constituents of the drought-stressed samples were higher than those without OPWV (Figure 5A–D).

### 2.4. Hydrogen Peroxide, Osmolyte and Lipid Peroxidation of OPWV-Treated Pandanus amaryllifolius

**Figure 6:**
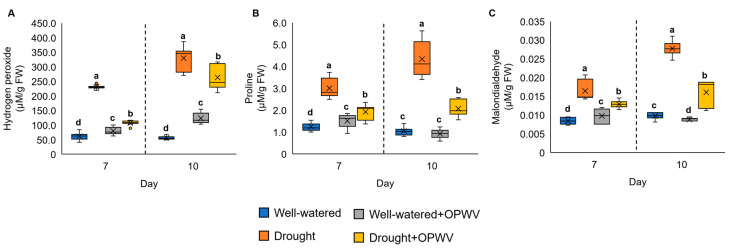
The hydrogen peroxide (H_2_O_2_), proline and malondialdehyde (MDA) contents in the well-watered and drought-stressed *Pandanus amaryllifolius* plants with or without OPWV for 7 and 10 days. (**A**) H_2_O_2_. (**B**) Proline. (**C**) MDA. The letter labelled on the mean value indicates a significant level between treatments based on the one-way ANOVA, followed by the post hoc test when its *p*-value < 0.05. The letter ‘a’ above the bars indicates the highest value, followed by ‘b’, ‘c’, and ‘d’. The (×) labelled in the box plot indicates the mean value of the treatment, while the (•) refers to the outlier value of the replicates.

In this experiment, the drought-stressed *P. amaryllifolius* on both days 7 and 10 showed a significant increment in hydrogen peroxide (H_2_O_2_), proline and MDA compared to the well-watered samples (Figure 6A–C). In contrast, applying OPWV to the drought-stressed *P. amaryllifolius* reduced the content of H_2_O_2_, proline and MDA (Figure 6A–C).

### 2.5. Antioxidant Enzymes Altered in Pandanus amaryllifolius in Response to OPWV and Drought Stress

**Figure 7:**
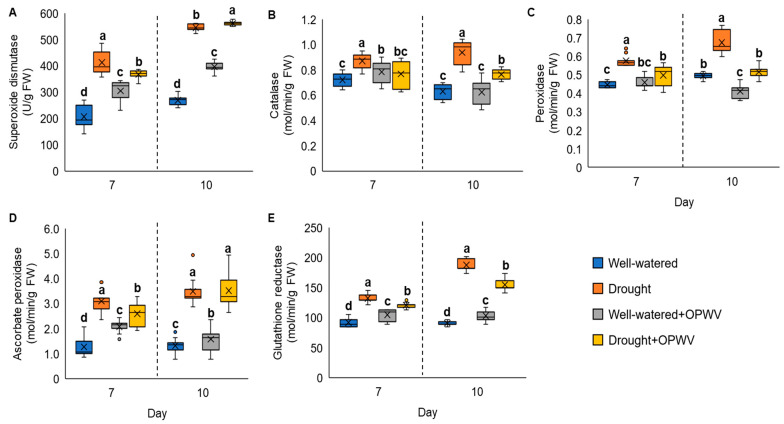
The antioxidant enzyme activities of the well-watered and drought-stressed *Pandanus amaryllifolius* plants with or without OPWV for 7 and 10 days. (**A**) Superoxide dismutase (SOD). (**B**) Catalase (CAT). (**C**) Peroxidase (POD). (**D**) Ascorbate peroxidase (APX). (**E**) Glutathione reductase (GR). The letter labelled on the mean value indicates a significant level between treatments based on the one-way ANOVA, followed by the post hoc Tukey test when its *p*-value < 0.05. The letter ‘a’ indicates the highest value, followed by ‘b’, ‘bc’, ‘c’ and ‘d’. The (×) labelled in the box plot indicates the mean value of the treatment, while the (•) refers to the outlier value of the replicates.

Drought stress induced the antioxidant enzyme activities of *P. amaryllifolius* on both days 7 and 10 (Figure 7). However, after being treated with OPWV, the superoxide dismutase (SOD), catalase (CAT), peroxidase (POD), ascorbate peroxidase (APX) and glutathione reductase (GR) activities of the drought-stressed samples were significantly reduced (Figure 7A–E), except for SOD and APX activities on day 10. Interestingly, applying OPWV to well-watered Pandanus plants increased their SOD, APX and GR activities (Figure 7A,D,E).

### 2.6. Pandanus amaryllifolius Drought-Stressed Responsive Genes Triggered with OPWV Application

**Figure 8:**
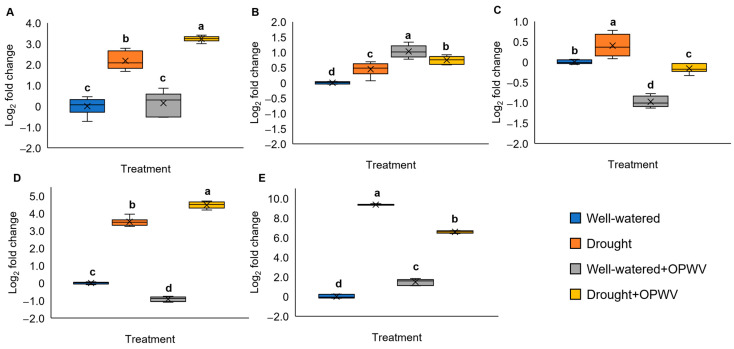
The drought-responsive gene expression of the well-watered or drought-stressed *Pandanus amaryllifolius* treated with or without OPWV for 7 days. (**A**) *Heat shock protein 70* (*PaHSP70*). (**B**) *Glyceraldehyde-3-phosphate dehydrogenase* (*PaGAPDH*). (**C**) *Enolase* (*PaENO*). (**D**) *Thaumatin* (*PaThau*). (**E**) β-fructofuranosidase (Paβ-Fruc). Actin and elongation factor-1 of *P. amaryllifolius* were reference genes for gene expression normalization. The letter labelled on the mean value indicates a significant level between treatments based on the one-way ANOVA, followed by the post hoc Tukey test when its *p*-value < 0.05. The letter ‘a’ indicates the highest value, followed by ‘b’, ‘c’, and ‘d’. The (×) labelled in the box plot indicates the mean value of the treatment, while the (•) refers to the outlier value of the replicates.

The expression of several drought-responsive genes was determined to understand the OPWV effects on *P. amaryllifolius* under different conditions. These genes were selected based on our previous findings [18]. Overall, all gene expressions were significantly up-regulated on 7-day drought stress (Figure 8A–E). However, the OPWV-treated drought-stressed plants showed increased *PaHSP70*, *PaGAPDH* and *PaThau* expression (Figure 8A,B,D) but decreased expression for PaENO and Paβ-Fruc (Figure 8C,E). In contrast, applying OPWV to the well-watered plants increased the expression of *PaHSP70*, *PaGAPDH*, and *Paβ-Fruc* (Figure 8A,B,E) but decreased *PaENO* and *PaThau* expressions (Figure 8C,D).

### 2.7. OPWV GC-MS Profiling

GC-MS analysis was conducted to profile the compounds present in the OPWV diluent of 1:500. In total, there are 25 compound peaks identified in the 25 min GC-MS run (Figure S3). As shown in Table 1, the OPWV major component was phenyl carbamate (64%), with other minor components of phenol (16%), guaiacol (4%) and syringol (3%).

## 3. Discussion

### 3.1. OPWV Induces Morphological Alteration and Improves Drought Tolerance

Drought affects plant growth and development. Plants adapt to such detrimental conditions by exploiting their complex morpho-physiological, biochemical and molecular mechanisms [20]. In this study, the imposed drought stress negatively affected the plant growth parameters, such as LRWC, leaf yellowing and folding, stem circumference, and root-to-shoot ratio. Conversely, spraying OPWV on plants prior to drought stress improved their tolerance by increasing LRWC and reducing REL, leaf wilting and leaf yellowing. This finding suggests that the OPWV-treated plants have better cell water holding capacity and structural integrity, thus enhancing their survivability under drought conditions. Plants with a lesser or slower leaf wilting are crucial for maintaining their yield and drought tolerance [21]. Similar findings have been shown in wheat, banana, and Arabidopsis when primed with wood vinegar, sodium nitroprusside and seaweed extract, respectively [17,22,23]. Although the exact mechanism is unclear, we speculate that the improved growth might be due to the reduced leaf folding and wilting by OPWV.

### 3.2. OPWV Ameliorated Photosynthetic Pigments

Photosynthesis is crucial in providing material and energy for plant growth, yet it is very susceptible to environmental stress. Generally, drought stress reduces the plant photosynthesis rate due to damage that occurs to its machinery, such as chlorophyll pigments [24,25]. In this study, OPWV treatment induced a higher accumulation of total chlorophyll content, including chlorophyll *a* and *b*, in either well-watered or drought-stressed plants. Our results correspond to the study by Vannini et al., where sweet chestnut wood distillate-primed lettuce plants recorded higher chlorophyll content than non-treated samples [16]. In addition, another study showed that rapeseed plants primed with peach wood vinegar before being exposed to salt stress produced higher chlorophyll content than salt-stressed non-primed rapeseed [26]. These findings evince the potential of OPWV as a biostimulant to boost and maintain plant photosynthesis machinery, even under stress conditions.

### 3.3. OPWV Mitigated Drought Stress Indicators

Drought stress induces oxidative damage to plants. Over-accumulation of ROS species, such as H_2_O_2_, causes damage to cellular molecules, such as lipids, and alters intrinsic membrane properties. MDA, a product formed via lipid peroxidation of arachidonic acid and large polyunsaturated fatty acid (PUFA) [27], is often used as a marker to indicate cell damage due to ROS. In this study, the drought-stressed plants exhibited increased H_2_O_2_ and MDA levels but were reduced with OPWV application, suggesting OPWV could mitigate ROS accumulation and cell damage. Other studies showed similar findings. For instance, the wheat and carrot plants treated with H_2_O_2_ as well as sodium nitroprusside [28] and α-tocopherol [29], respectively, showed lower ROS and MDA accumulation than the control. Interestingly, the well-watered *P. amaryllifolius* treated with OPWV showed increased H_2_O_2_ and MDA. This might be due to a sudden boost of H_2_O_2_, causing damage to the cell’s lipids and increased MDA. A similar finding has been reported by Gohari et al., where the authors found that biostimulant-treated well-watered basil plants had higher H_2_O_2_ and MDA levels than the control [30].

Proline, a free amino acid, is often used as an indicator of stress. Under normal conditions, proline biosynthesis predominantly occurs in the cytosol, while an imbalance of osmotic pressure in the cell during drought stress causes proline production in both cytosol and chloroplast, which preferred the glutamate pathway proline metabolism instead of the ornithine pathway [31]. In this study, a significant build-up of proline in drought-stressed *P. amaryllifolius* was observed. However, plants treated with OPWV prior to the drought stress experienced lower proline accumulation. This finding was in agreement with previous studies [32,33]. For instance, foliar silicon treatment on salt-stressed-sweet pepper showed reduced proline accumulation [34]. Furthermore, drought-stressed sugar beet recorded lower proline content when treated with silicone and proline [35]. Although the biostimulatory mechanisms of OPWV are unclear, this finding implies that OPWV could alleviate the adverse effects of drought stress.

### 3.4. Enhanced Antioxidant Responses with OPWV Application

To counteract the harmful effects of ROS, plants have developed antioxidant defense mechanisms that include enzymatic components. When stressed, plants may produce excessive levels of ROS, which in turn increases the activity of antioxidant enzymes. This investigation observed increased antioxidant activities in drought-stressed *P. amaryllifolius* plants, indicating the plants were coping with the stress by scavenging harmful ROS. The initial line of defense in the antioxidant defense system appears to be provided by an increase in the activity of SOD, an enzyme that converts superoxide anion into H_2_O_2_. The increase in SOD activity under drought stress conditions is consistent with several previous observations [28,35]. An increase in the activity of CAT, APX, POD and GR by drought treatment indicates their role in H_2_O_2_ detoxification. CAT, POD and APX convert H_2_O_2_ into water and oxygen with the help of GR.

On the other hand, OPWV treatment reduced the antioxidant activities, such as SOD, CAT, POD, APX, and GR, in drought-stressed *P. amaryllifolius*. This finding was in contrast with other previous studies. For instance, applying diluted honey to fava beans enhanced their antioxidant activities in both well-watered and drought conditions [36]. It is noteworthy that OPWV treatment increased SOD, CAT, APX, and GR on well-watered *P. amaryllifolius*. Similar results have been reported by Zhu et al., where rapeseed plants primed with poplar-derived wood vinegar showed increased antioxidant activities [15]. Taken together, our findings suggest that priming plants with OPWV might activate their antioxidant defense mechanism, and OPWV-primed plants could overcome the drought stress effect since they recorded decreased antioxidant activities.

### 3.5. Carbohydrate-Related and Drought-Responsive Genes Altered after OPWV Treatment

Several carbohydrate-related, such as *PaGAPDH*, *PaENO* and *Paβ-Fruc*, and drought-responsive gene expressions, such as *PaHSP70* and *PaThau*, were determined. As expected, the drought-stressed *P. amaryllifolius* showed increased expression of *PaHSP70*, *PaENO*, *PaThau*, *PaGAPDH*, and *Paβ-Fruc* compared to well-watered samples. It is unsurprised that plants respond to stressors by activating their stress defense and carbohydrate-related genes to improve their survival. Increased carbohydrate metabolism is needed to meet the energy demands in drought tolerance. These findings were aligned with previous studies [37,38,39].

*HSP70* and *Thau* genes have been shown to play an important role in plants responding to environmental stresses, such as heat and drought stress. Heat shock proteins (HSPs) are molecular chaperones that play a crucial role in maintaining proper protein folding and preventing aggregation in plants. These processes are vital for cell survival under stress conditions, such as drought. Several members of the *HSP70* gene family have been identified in various plant species. Some of these *HSP70* members have been reported to be involved in the plant’s response to drought stress. For example, research has shown that the expression of *HSP70* in soybean plants increases in response to drought stress. On the other hand, thaumatin is a group of small and highly hydrophilic proteins found in various plant species. These proteins are involved in various plant stress responses, including drought stress. For instance, the drought-stressed durum wheat plants revealed an increased *Thau*, suggesting its vital role in the plant’s adaptive response to drought stress [40].

Applying OPWV, however, reduced the expression of *PaENO* and *Paβ-Fruc*. Although the exact mechanism is unclear, the downregulation of these genes did not affect the overall plant tolerance against drought stress. On the contrary, *PaGAPDH*, *PaHSP70* and *PaThau* showed elevated expression in the OPWV-treated drought-stressed samples. In addition, several studies showed that *GAPDH*, *HSP70* and *Thau* play an essential role in plant biotic and abiotic tolerances [41,42,43,44]. These findings suggest that OPWV could modulate these drought-responsive genes to improve plant drought tolerance.

### 3.6. OPWV Compound Profile Elucidated

Wood vinegar is a complex mixture of water and various organic compounds, including acids, alcohols, phenols, aldehydes, and esters [45]. However, the composition of wood vinegar can vary depending on factors such as the pyrolysis process employed, the moisture content of the feedstock, and the type of biomass used, although differences in this last case are usually scarce. In this study, we identified 16 different compounds in OPWV. Of these, phenyl carbamate or anthranilic acid (C_7_H_7_NO_2_), phenol, syringol, guaiacol, catechol, and tetrasiloxane were the major constituents in OPWV. These compounds have been reported in other wood vinegar [11,46,47] and have shown health-beneficial properties. For instance, phenyl carbamate, syringol, guaiacol, catechol, and tetrasiloxane exhibited antioxidant properties [12,13,48,49], which might be necessary for drought tolerance in plants. High phenols and their derivatives content in wood vinegar originated from the pyrolytic degradation of lignin. During the thermal reaction, lignin degradation releases aromatic compounds, such as guaiacyl and syringyl, resulting from the cleavage of alpha-ether and beta-ether weak bonds. Lignin is typically converted into derivatives, such as phenol, syringol, and catechol. Other chemical compounds present in wood vinegar, including alcohols, aldehydes, ketones, carboxylic acids, and ethers, are primarily derived from the decomposition of cellulose and hemicelluloses. Studies have also shown that wood vinegar contains high phenolic compounds, indicating its potential use as an antioxidant agent [11,47]. Since phenolic compounds are the largest group in OPWV, we speculate that adding them to plants might help them cope with drought stress, as shown in reduced antioxidant enzyme activities in OPWV-treated plants. However, how plants interact with these compounds or does this involve their intrinsic antioxidant systems remains unknown.

The improved plant morphological structure, such as root biomass, by OPWV might also be due to the compounds in OPWV. For example, anthranilic acid is an early precursor of auxin indole-3-acetic acid (IAA) in root cells [50]. Therefore, applying OPWV-containing anthranilic acid on Pandanus leaves could probably induce IAA synthesis, which is critical for root formation. On the other hand, syringol and guaiacol, the main components of lignin in OPWV, might improve the plant cell structural integrity and induce lignin biosynthesis [51].

## 4. Materials and Methods

### 4.1. Plant Material

Three-month-old uniform-size and disease-free *P. amaryllifolius* plants obtained from Green Nursery Sdn. Bhd., Muar, Johor, Malaysia, were transferred to polybags (20 × 11 cm^2^) consisting of 600 g clay slit loam soil and acclimatized for 2 weeks in a growth room at Universiti Malaya, Malaysia. Whilst acclimatizing, the plants were treated with foliar fertilizer, Mr Garnick 20X (Baba, Pulau Pinang, Malaysia) once a week. All plants were maintained at 28 ± 2 °C, under a relative humidity of 80 ± 5% and 1500 lux light intensity for a 12:12 h photoperiod cycle. About 20 mL of distilled water was supplemented to all plants once a day.

### 4.2. Experimental Design and Foliar Application

**Figure 9:**
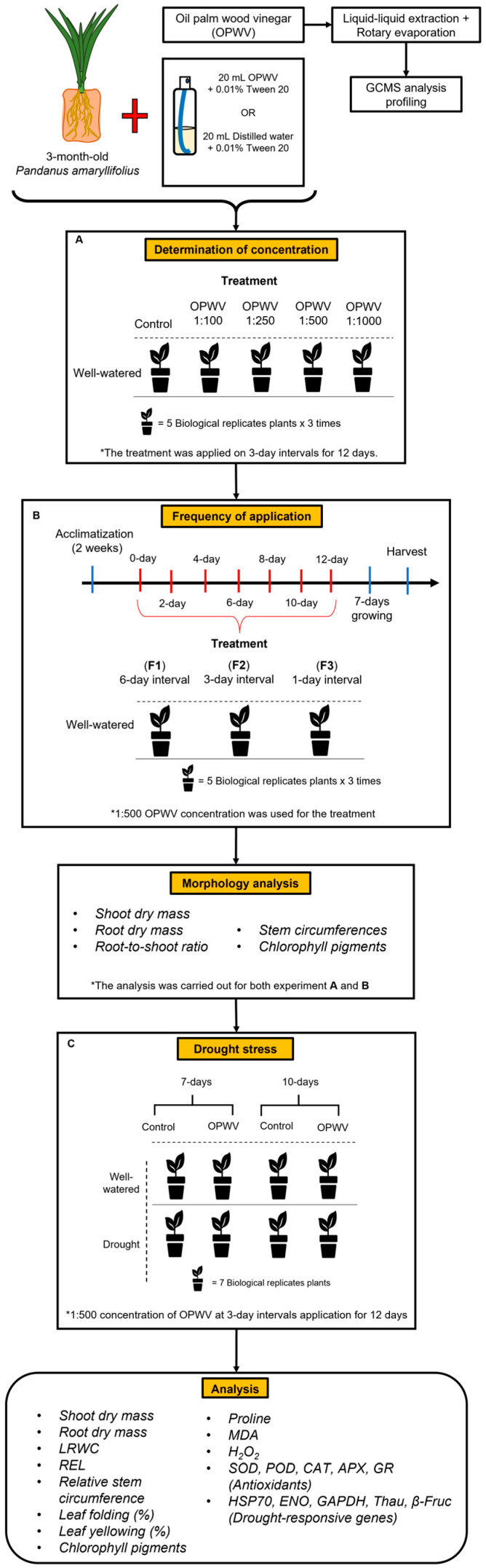
The schematic diagram of the experimental setup. (**A**) *Pandanus amaryllifolius* plants were sprayed with oil palm wood vinegar (OPWV) at 1:100, 1:250, 1:500, and 1:1000 dilutions in 3-day intervals until 12 days. The plants were allowed to grow for an additional 7 days before harvesting. (**B**) *P. amaryllifolius* plants were sprayed with OPWV at 1:500 dilution at 6-day intervals (F1), 3-day intervals (F2), and 1-day intervals (F3). Well-watered plants served as control. (**C**) The optimized concentration and application frequency of OPWV was used to determine its potential to mitigate the drought stress effects. *P. amaryllifolius* plants were well-watered or treated with 7- or 10-day drought stress with or without OPWV.

OPWV obtained from Palm Leaf Trader Sdn. Bhd., Malaysia, was diluted to 1:100, 1:250, 1:500, and 1:1000 with distilled water containing 0.01% (*v*/*v*) Tween-20. These dilutions were selected based on the manufacturer’s recommendations. The diluted OPWV (20 mL) was applied to each plant using a small handheld sprayer (Figure S2A) on the leaves adaxially and abaxially until runoff. After being air-dried, the plants were randomly placed in the growing plot. Foliar application of OPWV was repeated at 3-day intervals until 12 days (Figure 9A). The plants were allowed to grow for an additional 7 days before harvesting (Figure 9A,B). Distilled water containing 0.01% Tween-20 served as control. All plants received 20 mL of distilled water daily. Each treatment consisted of 5 plants, and the experiment was repeated thrice.

To determine the frequency of OPWV application, the determined diluted OPWV was applied to *P. amaryllifolius* plants once at 6-day intervals (F1), 3-day intervals (F2), or 1-day intervals (F3) using a similar method, as previously described (Figure 9B). Distilled water containing 0.01% Tween-20 served as control. All plants received 20 mL of distilled water daily. Each treatment consisted of 5 plants, and the experiment was repeated thrice.

The plant morphological changes for both experiments, such as DW, root-to-shoot ratio, and relative stem circumference, were recorded, and chlorophyll content was measured as described below.

### 4.3. Determination of Photosynthetic Pigment Contents

Chlorophyll pigments were measured according to Lichtenthaler [52]. The fresh leaves were ground and freeze-dried overnight to remove water content in the cells prior to the pigment’s extraction. About 100 mg of the lyophilized leaf powder was added with 2 mL 90% (*v*/*v*) acetone in a 2 mL centrifuge tube. The mixture was kept in the dark for about 20 min before centrifugation at 10,000× *g* for 15 min to collect the supernatant. Then, 100 µL of the supernatant was diluted with 900 µL 80% (*v*/*v*) acetone before the absorbance of the mixture was measured using a spectrophotometer, NanoPhotometer P300 (Implen GmbH, Munich, Germany) at 470, 647, and 663 nm wavelengths. Each sample was analyzed in triplicate. The chlorophyll and carotenoid contents were calculated as follows:

Chlorophyll *a* (µg mg^−1^) = 12.25A_663_ − 2.79A_647_ (1)

Chlorophyll *b* (µg mg^−1^) = 21.50A_647_ − 5.10A_663_ (2)

Total chlorophyll (µg mg^−1^) = 7.15A_663_ + 18.71A_647_ (3)

Carotenoids (µg mg^−1^) = (1000A_470_ − 1.82C*_a_* − 85.02C*_b_*)/198 (4)

C*_a_* = Chlorophyll *a*
C*_b_* = Chlorophyll *b*

### 4.4. Drought Treatment

*P. amaryllifolius* plants were well-watered or treated drought stress with or without OPWV at 1:500 dilution. The OPWV was foliar applied to *P. amaryllifolius* plants at 3-day intervals (F2). Drought treatment was conducted by withholding water for 7 or 10 days, according to Amnan et al. [18]. After drought treatment, each plant’s root and shoot DW, root-to-shoot ratio, and relative stem circumference were recorded (Figure 9C). Chlorophyll content was measured as previously described. The leaf numbers 3, 4, and 5 were harvested and wiped with distilled water and tissue paper before being used for LRWC, REL, gene expression and biochemical analyses to ensure consistency and standardization of data recording. Each treatment consisted of 7 independent biological replicates (three technical replicates within each) (Figure S2B). The overall experimental design is shown in Figure 9.

### 4.5. Leaf Relative Water Content

The harvested leaf samples were immediately processed to measure the LRWC, according to Turner [53]. In brief, the leaf samples were wiped with 70% ethanol and cut into several pieces (2 cm^2^) prior to fresh weight (FW) weighing. The cut leaf pieces were then transferred into a Petri dish and submerged in 20 mL of distilled water for 6 h at room temperature. After being submerged, the saturated weight (SW) of the leaf pieces was measured before being subjected to oven drying for 2 days. The DW of the leaf samples was then measured, and LRWC was calculated using the following formula:

LRWC (%) = [(FW − DW)/(SW − DW)] × 100(5)

### 4.6. Relative Electrolyte Leakage

The REL was analyzed according to Quan et al. by immersing 100 mg leaves pieces in 10 mL deionized water before incubating at room temperature for 6 h under shaking conditions of 150 rpm [54]. Initial electrical conductivity (C_i_) was measured using a conductivity meter, Cyberscan CON 11 (Thermo Fisher Scientific Inc., Waltham, MA, USA) before boiling the leaf samples for 20 min. The conductivity of lysed cells (C_max_) was then measured. The REL was calculated using the following formula:

REL (%) = C_i_/C_max_ × 100 (6)

### 4.7. Plant Weight and Relative Stem Circumference

The fresh weight of the plants was analyzed immediately after harvesting, whereby the shoots and roots were separately weighed. The plants were collected and stored in a paper bag before drying in an oven for at least 7 days or until a constant weight was achieved to measure the DW. The root-to-shoot ratio was then calculated by dividing the DW of roots and shoots.

The stem circumference of each plant was recorded on days 0, 12 (end of foliar application treatment), 19 (end of the 7-day drought), and 22 (end of the 10-day drought) to analyze and compare the relative stem circumference between each treatment. The stem circumference was measured at the meeting point between the shoot and stem using a flexible one-meter measuring tape (Figure S2C).

### 4.8. Examination of Folded and Yellowing Leaves

Throughout the experiment, the leaf shape and color of *P. amaryllifolius* were observed and recorded. The leaf shape was categorized into three groups: No folding (Normal), less than 50% folding (Stage 1), and more than 50% folding (Stage 2) (Figure S2D). The number of folded leaves per plant was calculated accordingly to its stage as follows:

Percentage of folding leaves (%) = (Number of folded leaves per plant/Total number of leaves per plant) × 100(7)

The leaf color was recorded based on the number of yellowing leaves per plant according to the yellowing percentage scale (Figure S1E). The leaves with a 25% yellowing area and above were considered yellowing. The number of yellowing leaves per plant was calculated as follows:

Percentage of yellowing leaves (%) = (Number of yellowing leaves per plant/Total number of leaves per plant) × 100(8)

### 4.9. Malondialdehyde Content

MDA level in the leaf samples was analyzed according to Heath and Packer [55]. About 100 mg of fresh leaves were ground into a fine powder in a mortar with 1.5 mL 0.1% trichloroacetic acid (TCA) and transferred into a 2 mL centrifuge tube. The ground sample was centrifuged at 13,000× *g* for 10 min at 4 °C. The supernatant (300 µL) was mixed with 1 mL mixture containing 0.5% (*v*/*v*) thiobarbituric acid (TBA) in 20% (*w*/*v*) TCA. The mixture was heated at 95 °C for 30 min, ice-cooled, and centrifuged at 10,000× *g* for 10 min. The MDA content was calculated with the formula below from the absorbance measurement of the supernatant at 532 and 600 nm wavelengths using a spectrophotometer, NanoPhotometer P300 (Implen GmbH, Munich, Germany).

MDA = [(A_532_–A_600_) × VTr × 1000]/(Extinction coefficient MDA × 1 cm × Ve)/g FW (9)

A_532_–A_600_ = Absorbance of MDA–TBA
VTr = Volume of reaction (mL)
Ve = Volume of enzyme extract (mL)

The extinction coefficient of this MDA-TBA abduct at 532 nm is 155 mM^−1^ cm^−1^.

### 4.10. Proline Content

Proline content was determined, as described by Bates et al. [56]. About 2 mL 70% (*v*/*v*) ethanol was added to 200 mg lyophilized leaf powder. After grinding, the mixture was centrifuged at 13,000× *g* for 20 min. The collected supernatant was mixed with 500 µL sample extract or proline standards (500 µL glacial acetic acid: 500 µL freshly prepared acid-ninhydrin reagent). The mixture was vortexed and boiled at 100 °C in a heat block for 1 h and cooled in ice for 30 min. The pigmentation developed during the reaction was extracted by adding 1 mL toluene, vortexed and centrifuged at 13,000× *g* for 20 min. The toluene phase was carefully collected into a new centrifuge tube before being measured at a 520 nm wavelength with toluene as a blank. A series of proline standards with the concentration of 3, 5, 10, 25, 50, 75, and 100 µM were measured to construct a proline standard curve which was then used to calculate the proline content in the leaf samples based on the following formula:

Proline (µM g^−1^ FW) = [(μg proline/mL × mL toluene)/μg 115.5/μmole]/(g FW/5) (10)

### 4.11. Antioxidant Enzyme Assays

The leaf samples were extracted in an extraction buffer containing (100 mM phosphate buffer, pH 7.0, 0.1 mM disodium ethylenediaminetetraacetic acid, and 0.1 g polyvinylpyrrolidone). About 200 mg of lyophilized leaf powder was mixed with 2 mL of the cold extraction buffer before centrifuging at 13,000× *g* for 10 min at 4 °C. The supernatant was collected and used for the subsequent antioxidant enzymatic assays. The enzyme activity for each assay was calculated using the formula below:

Enzyme activity (M min^−1^ g^−1^ FW) = (∆A× VTr)/(ε × ∆t × 1 cm × Ve × g FW) × 1000 (11)

∆A = Difference in absorbance
VTr = Volume of reaction (mL)
Ve = Volume of enzyme extract (mL)
∆t = Difference in time of absorbance (min)
For CAT, ε(Hydrogen peroxide) = 36.0 mol^−1^ cm^−1^
For APX, ε(Ascorbic acid) = 2.8 mmol^−1^ cm^−1^
For POD, ε(Tetraguaiacol) = 26.6 mol^−1^ cm^−1^
For GR, ε(NADPH) = 6220 mol^−1^ cm^−1^

#### 4.11.1. Catalase

CAT activity in the leaf sample was analyzed, as described by Aebi with minor modifications [57]. The 3 mL reaction mixture comprised 50 mM phosphate buffer (pH 7.0), freshly prepared 8.33 mM H_2_O_2_, and 100 µL enzyme extract, which was added last to initiate the reaction. The enzyme activity was monitored and measured at 240 nm for 2 min with a 15 s reading interval.

#### 4.11.2. Ascorbate Peroxidase

The APX assay was determined by Chen and Asada [58]. The 1 mL reaction mixture consisted of 50 mM phosphate buffer (pH 7.0), 200 µL enzyme extract, 0.5 mM ascorbic acid, and 0.42 mM H_2_O_2_. H_2_O_2_ was added last to initiate the reaction. The enzyme activity was monitored and measured at 290 nm for 2 min with a 15 s reading interval.

#### 4.11.3. Peroxidase

POD activity was analyzed as described by Chance and Maehly with minor modifications [59]. The 1 mL reaction mixture contained 100 mM phosphate buffer (pH 7.0), 0.5 mM guaiacol, 0.0833 mM H_2_O_2_, and 100 µL enzyme extract. H_2_O_2_ was added last to initiate the reaction. The POD activity was measured at 470 nm wavelength for 2 min with a 15 s reading interval.

#### 4.11.4. Glutathione Reductase

GR assay was conducted according to Mannervik [60]. The reaction mixture comprised of 500 µL assay buffer (0.2 M potassium phosphate buffer, pH 7.0, 0.2 mM EDTA), 50 µL 20 mM freshly prepared oxidized glutathione, 50 µL 2 mM NADPH solution, distilled water, and 300 µL enzyme extract. The decrease in absorbance at 340 nm after adding enzyme extract was monitored for 1 min with a 15 s reading interval.

#### 4.11.5. Superoxide Dismutase

According to Dhindsa et al., the SOD activity in the sample was determined by a 3 mL reaction mixture containing 50 mM phosphate buffer (pH 7.0), 9.9 mM L-methionine, 55 µM nitro blue tetrazolium, 0.025% (*v*/*v*) Triton X-100 (Thermo Fisher Scientific, Inc., Waltham, MA, USA), 100 µL enzyme extract, and 4.8 µM riboflavin [61]. This reaction was prepared in an aluminium-covered test tube. The reaction was initiated by adding the riboflavin, mixing by shaking, and incubating at 30 °C for 10 min under a white light source (35 W) placed at 20 cm height above the test tubes. After the reaction, the mixture was measured at 560 nm with a blank prepared using the extraction buffer to replace the sample. The SOD activity was calculated based on the formula below:

SOD (Unit g^−1^ FW) = [(Blank − Sample) A_560nm_/(Blank A_560nm_)] × (Volume reaction)/(Volume enzyme) × 100 × 1/50/0.1 g FW (12)

#### 4.11.6. Hydrogen Peroxide

H_2_O_2_ content was analyzed according to Velikova et al. with some modifications [62]. In an ice-cold mortar, 100 mg of leaf powder was homogenized with 1.5 mL 0.1% (*w*/*v*) TCA and transferred into a tube. The mixture was centrifuged at 10,000× *g* for 15 min at 4 °C. About 250 µL of the collected supernatant was mixed with a 1 mL reaction mixture containing 2.5 mM potassium phosphate buffer, pH 7.0, and 0.5 M potassium iodide. The H_2_O_2_ level was calculated based on the reaction absorbance of the leaf samples and H_2_O_2_ standards of 2.5 to 100 µM measured at 390 nm wavelength.

### 4.12. RNA Extraction and cDNA Synthesis

The RNA was extracted using the cetyltrimethylammonium bromide conventional method, according to Asif et al. [63]. Briefly, 100 mg of finely ground leaf powder was used to extract the RNA. The final pellet was eluted in 30 µL of nuclease-free water to determine its concentration (ng/µL) and purity at A260/280 and A260/230 wavelengths using a spectrophotometer, NanoPhotometer P300 (Implen GmbH, Munich, Germany). Next, the extracted RNA was treated with DNase by RapidOut DNA removal kit (Thermo Scientific) to remove genomic DNA, followed by an RNA precipitation step. The precipitated DNA-free and DNase-free RNA was air dried before eluting with 30 µL of RNase-free water before measuring its concentration with a spectrophotometer. The RNA was then converted to cDNA using an NxGen M-MuLV Reverse Transcriptase (Lucigen) following the manufacturer’s protocol. The RNA samples were stored at −80 °C until further use.

### 4.13. Quantitative Real-Time PCR

Quantitative real-time PCR (qPCR) was performed to analyze the expression of drought-stressed responsive genes (Table 2). The qPCR consisted of a reaction volume of 10 μL containing 40 ng of cDNA, 0.2 µM primers, and 1× SG Fast qPCR Master Mix (Sangon Biotech Co., Ltd., Shanghai, China), with the elongation factor-1 and actin as reference genes (Table 2). The qPCR assay was conducted according to the manufacturer’s protocol. The relative expression levels were calculated according to Pfaffl [64]. The qPCR analysis was conducted with three biological replicates and three technical replicates for each gene.

### 4.14. Oil Palm Wood Vinegar (OPWV) Gas Chromatography-Mass Spectrometry (GC-MS) Profiling

The liquid–liquid extraction method using dichloromethane (DCM) was used to extract OPWV compounds [66,67]. Briefly, 50 mL of the crude OPWV was extracted with 50 mL DCM in a separating funnel. The organic layer was collected, whereas the aqueous layer was extracted twice with DCM. All organic layers were combined to a final volume of 150 mL while the aqueous layer was discarded. The solvent was removed using a rotary evaporator at 40 °C for 1 h. The dried residue was weighed and reconstituted in 10 mL methanol prior to GC-MS profiling. About 100 ppm of collidine was added to the methanol solution with a final volume of 1 mL. The mixture was then diluted at 1:30 with methanol before injecting into a GC-MS (Shimadzu Manufacturing Co., Ltd., Kyoto, Japan, QP-2010). The GC-MS analysis parameters were set as follows: capillary columns (SH-5MS); 30 m × 0.25 mm diameter × 0.25 µm thickness; a temperature of injection: 250 °C; column temperature program: 50–220 °C and helium flow rate: 4.7 mL/min. The GC-MS was arranged in the electron ionization mode at 70 eV with an ion source and interface temperature of 250 °C and 300 °C, respectively. About 1 μL of the sample was injected into a column and held at 50 °C for 2 min with an increasing rate of 8 °C/min until 250 °C at which the temperature was held for another 2 min. The compounds were identified by comparison with the standard library data calculated based on the integrated peak areas relative to the internal standard peak area.

### 4.15. Statistical Analyses

The morphology and biochemical assays data were analyzed by one-way analysis of variance (ANOVA) followed by a post hoc Tukey range using SPSS Statistics software (version 23.0; IBM). The analyzed data were considered statistically significant when its *p*-value < 0.05.

## 5. Conclusions

In summary, our investigation unveils the potential of OPWV as a biostimulant to mitigate drought stress in *P. amaryllifolius*. Applying OPWV at 1:500 dilution at 3-day intervals for 12 days improved growth parameters in *P. amaryllifolius*. Although the imposed drought decreased stem circumference, leaf structure and pigmentation, applying OPWV alleviated these adverse effects. Furthermore, increased ROS, proline and MDA contents, antioxidant activities and drought-responsive gene expression in drought-stressed plants were reduced by OPWV. In addition, several compounds in OPWV, such as phenyl carbamate or anthranilic acid, tetrasiloxane, syringol, guaiacol, and catechol, might be responsible for their biostimulant properties that have been identified. However, although OPWV showed beneficial effects to plant growth, further studies on its effects on open field conditions and/or other crops are desirable. Identifying each compound that gives such biostimulatory effects to plants might be helpful for product development.

## Figures and Tables

**Table 1:** GC-MS analysis of oil palm wood vinegar.

No.	Identified Compound	Molecular Formula	Retention Time (RT)	*m*/*z*	Area under Peak	Concentration (ppm/µL)	Concentration (ppm/mL)
1	Phenyl carbamate	C_7_H_7_NO_2_	7.111	94.15	48,765,158	340.55	340,550
2	Phenol	C_6_H_6_O	6.868	94.05	12,407,245	86.65	86,650
3 *	Pyridine, 2,4,6-trimethyl-	C_8_H_11_N	7.191	121.05	7,159,676	50.00	50,000
4	Guaiacol	C_7_H_8_O_2_	9.079	109.05	3,369,657	23.53	23,530
5	Syringol	C_8_H_10_O_3_	13.856	154.05	2,215,362	15.47	15,470
6	2-(2′,4′,4′,6′,6′,8′,8′-Heptamethyltetrasiloxan-2′-yloxy)-2,4,4,6,6,8,8,10,10-nonamethylcyclopentasiloxane	C_16_H_48_O_10_Si_9_	19.103	73.1	1,836,709	12.83	12,830
7	3-Isopropoxy-1,1,1,7,7,7-hexamethyl-3,5,5-tris(trimethylsiloxy) tetrasiloxane	C_18_H_52_O_7_Si_7_	23.366	73.1	1,741,464	12.16	12,160
8	Catechol	C_6_H_6_O_2_	11.458	110.05	1,096,505	7.66	7660
9	3-Oxabicyclo[3.3.0]oct-7-en-2-one,4-methoxy-	C_9_H_12_O_3_	8.946	109.05	961,798	6.72	6720
10	o-Creosol	C_7_H_8_O	8.271	108.05	803,567	5.61	5610
11	1,2-Cyclopentanedione, 3-methyl-	C_6_H_8_O_2_	8.011	112.05	753,407	5.26	5260
12	1,2,4-Trimethoxybenzene	C_9_H_12_O_3_	15.875	168.1	546,464	3.82	3820
13	4-Ethylguaiacol	C_9_H_12_O_2_	12.918	137.05	513,484	3.59	3590
14	3-Methoxyycatechol	C_7_H_8_O_3_	12.644	140.05	508,764	3.55	3550
15	Methoxyacetylene	C_10_H_14_O_3_	17.18	167.1	242,644	1.69	1690
16	Creosol	C_8_H_10_O_2_	10.938	123	190,595	1.33	1330
17	Methyl palmitate	C_17_H_34_O_2_	22.761	74.1	116,444	0.81	810

The asterisk indicates the internal standard spiked prior to injection.

**Table 2:** List of genes and their primer sequences used for relative expression analysis.

Gene	Sequences, 5′–3′
HSP70_Pandan (*PanHSP70*)	F-ACCTACAAGGGTGAGGAGAAG R-GAAATAGGCAGGGACAGTGATG
GAPDH_Pandan (*PanGAPDH*) [65]	F-AGGGTGGTGCCAAGAAGGT R-CCACCTCTCCAGTCCTT
Enolase_Pandan (*PanENO*)	F-TGAGTGATGGCACTTACGCC R-ACGTTCTCCACAGCCTTGAG
Thaumatin_Pandan (*PanThau*)	F-TCGCTGTCCTTCTCCTTTGG R-CACCTTGTGAGGAATGCAGC
β-fructofuranosidase_Pandan (*Panβ-Fruc*)	F-GAACCCTGGATGGTATCGGG R-CCGGCAAATGCTCCTAAGTG
* Actin_Pandan	F-GAGGCTATTCCTTCACCACTAC R-GTCTCAAGCTCCTCCTCATAATC
* Elongation factor-1_Pandan	F-TCTTCACAAAGCCAGCATCTC R-GACTGCCACACCTCTCATATTG

The asterisk (*) indicates the constitutive genes used as reference genes for gene expression normalization.

## Data Availability

The data presented in this study are available in Supplementary Materials.

## Supplementary Materials

The following supporting information can be downloaded at: https://www.mdpi.com/article/10.3390/plants12040785/s1, Figure S1. (**A**) Photographs of representative *Pandanus amaryllifolius* treated with 1:100, 1:250, 1:500, 1:1000 oil palm wood vinegar (OPWV) and distilled water (control) on days 0 and 19. (**B**) Photographs of representative *P. amaryllifolius* sprayed with OPWV at different frequencies on days 0 and 19. F1: applied at 6-day intervals, F2: applied at 3-day intervals, and F3: applied at 1-day interval. Bar = 10 cm; Figure S2. (**A**) Commercial handheld sprayer with 120 mL capacity. (**B**) The position of the harvested leaves. Leaf numbers 3, 4 and 5 were harvested for all assays. (**C**) The region for measuring the stem circumference. (**D**) Normal leaf and different stages of leaf folding. (**E**) The percentage of yellowing leaves from 0% to 100% yellowing. Figure S3. The GC-MS profile of oil palm wood vinegar (OPWV) at 1:500 dilution.
