# Supplementary material for: Foliar Application of Oil Palm Wood Vinegar Enhances Pandanus amaryllifolius Tolerance under Drought Stress
Source: Plants (Basel). 2023 Feb 9;12(4):785. doi: 10.3390/plants12040785 (PMC9958832; doi:10.3390/plants12040785)
Supplement: Supplementary file 1 [file plants-12-00785-s001.zip › plants-2166569-supplementary.pdf]

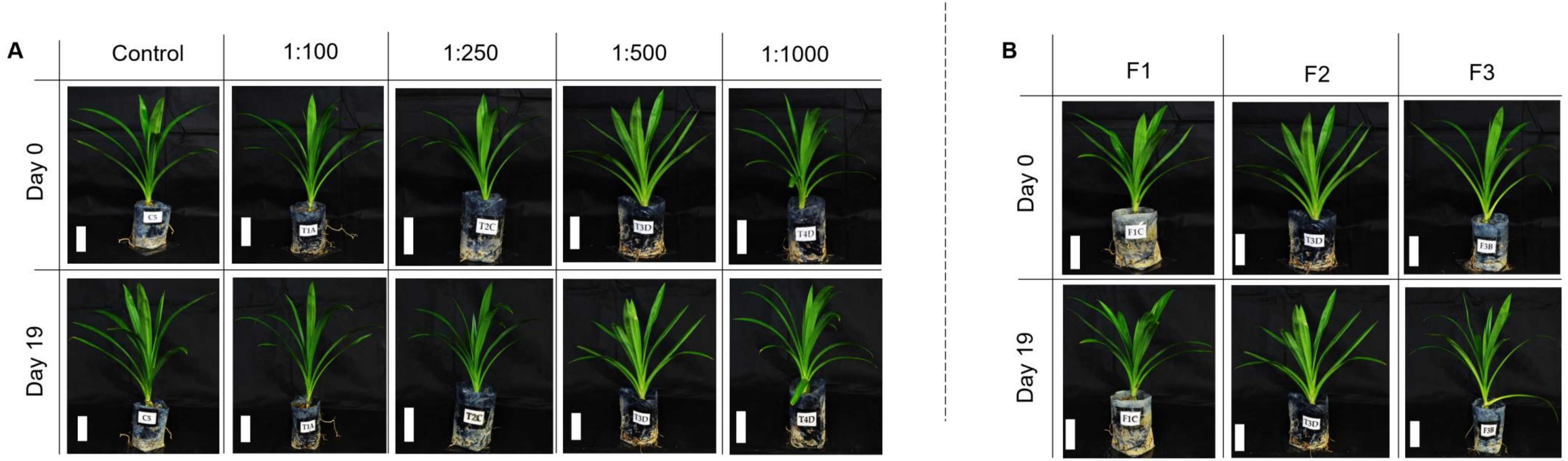


**Figure S1**. (**A**) Photographs of representative *Pandanus amaryllifolius* treated with 1:100, 1:250, 1:500, 1:1000 oil palm wood vinegar (OPWV) and distilled water (control) on days 0 and 19. (**B**) Photographs of representative *P. amaryllifolius* sprayed with OPWV at different frequencies on days 0 and 19. F1: applied at 6-day intervals, F2: applied at 3-day intervals, and F3: applied at 1-day interval. Bar = 10 cm.

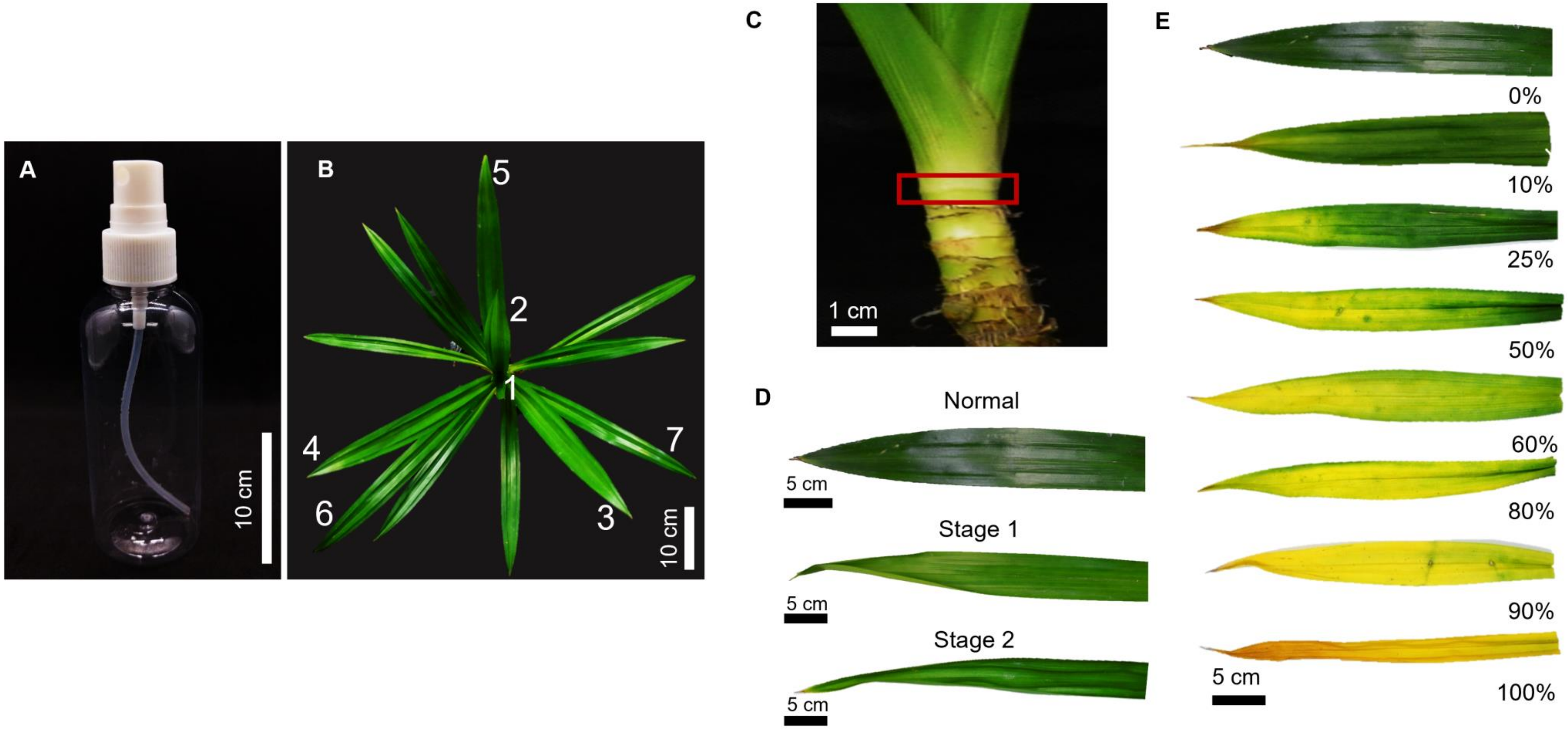


**Figure S2**. (**A**) Commercial handheld sprayer with 120 mL capacity. (**B**) The position of the harvested leaves. Leaf numbers 3, 4 and 5 were harvested for all assays. (**C**) The region for measuring the stem circumference. (**D**) Normal leaf and different stages of leaf folding. (**E**) The percentage of yellowing leaves from 0% to 100% yellowing.

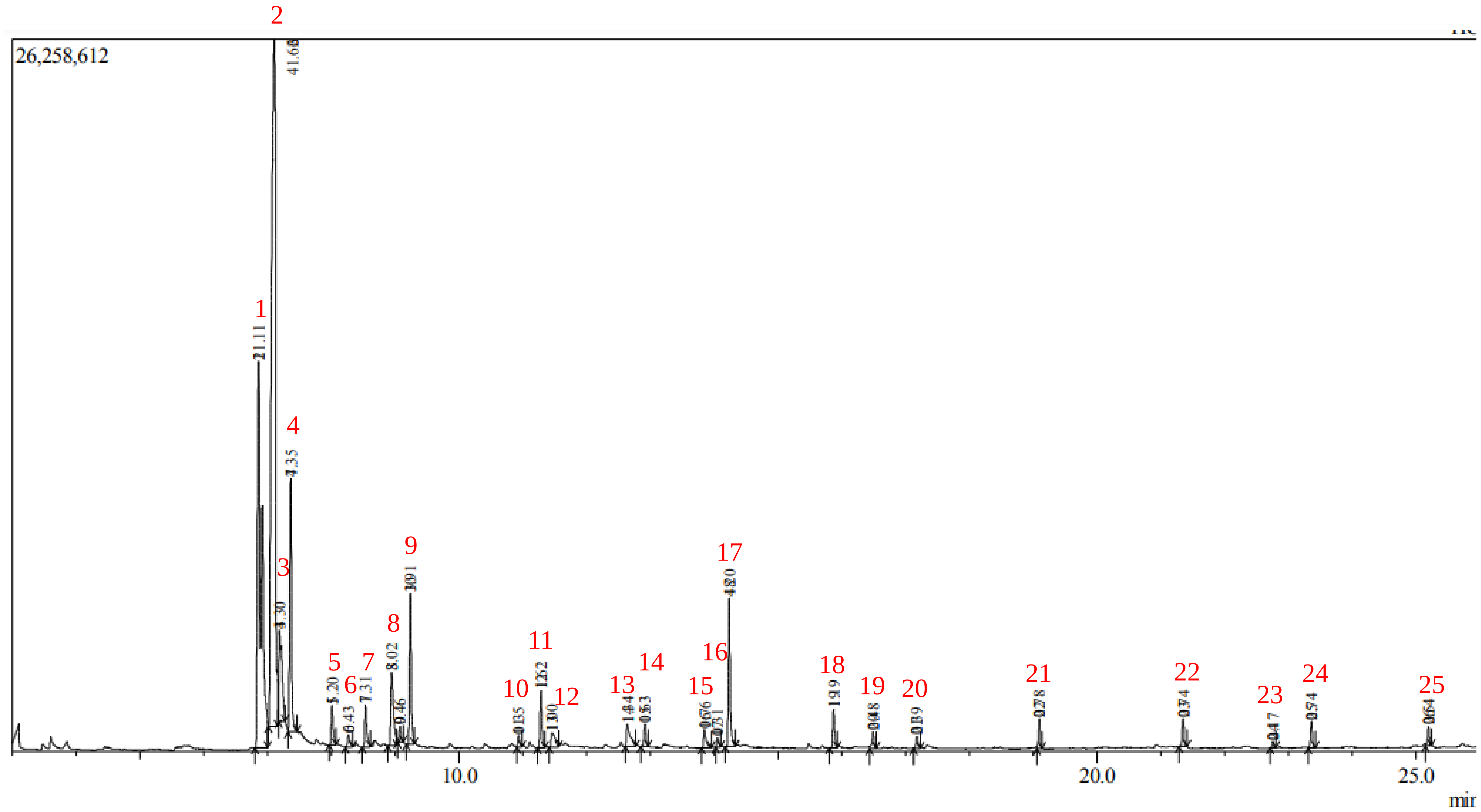


**Figure S3**. The GC-MS profile of oil palm wood vinegar (OPWV) at 1:500 dilution.
